# Exhaled Nitric Oxide in Systemic Sclerosis Lung Disease

**DOI:** 10.1155/2017/6736239

**Published:** 2017-02-14

**Authors:** Natalie K. Kozij, John T. Granton, Philip E. Silkoff, John Thenganatt, Shobha Chakravorty, Sindhu R. Johnson

**Affiliations:** ^1^University Health Network Pulmonary Hypertension Programme, Toronto General Hospital, Department of Medicine, University of Toronto, Toronto, ON, Canada; ^2^Department of Medicine, Temple University, Philadelphia, PA, USA; ^3^University Health Network Pulmonary Hypertension Programme, Toronto General Hospital, Toronto, ON, Canada; ^4^University Health Network Pulmonary Hypertension Programme, Toronto General Hospital, Toronto Scleroderma Program, Toronto Western Hospital, Mount Sinai Hospital, Department of Medicine, Institute of Health Policy, Management and Evaluation, University of Toronto, Toronto, ON, Canada

## Abstract

*Background*. Exhaled nitric oxide (eNO) is a potential biomarker to distinguish systemic sclerosis (SSc) associated pulmonary arterial hypertension (PAH) and interstitial lung disease (ILD). We evaluated the discriminative validity, feasibility, methods of eNO measurement, and magnitude of differences across lung diseases, disease-subsets (SSc, systemic lupus erythematosus), and healthy-controls.* Methods*. Consecutive subjects in the UHN Pulmonary Hypertension Programme were recruited. Exhaled nitric oxide was measured at 50 mL/s intervals using chemiluminescent detection. Alveolar and conducting airway NO were partitioned using a two-compartment model of axial diffusion (CMAD) and the trumpet model of axial diffusion (TMAD).* Results*. Sixty subjects were evaluated. Using the CMAD model, control subjects had lower median (IQR) alveolar NO than all PAH subjects (2.0 (1.5, 2.5) versus 3.14 ppb (2.3, 4.0), *p* = 0.008). SSc-ILD had significantly lower median conducting airway NO compared to controls (1009.5 versus 1342.1 ml⁎ppb/s, *p* = 0.04). SSc-PAH had increased median (IQR) alveolar NO compared to controls (3.3 (3.0, 5.7) versus 2.0 ppb (1.5, 2.5), *p* = 0.01). SSc-PAH conducting airway NO inversely correlated with DLCO (*r* −0.88 (95% CI −0.99, −0.26)).* Conclusion*. We have demonstrated feasibility, identified that CMAD modeling is preferred in SSc, and reported the magnitude of differences across cases and controls. Our data supports discriminative validity of eNO in SSc lung disease.

## 1. Introduction

Pulmonary arterial hypertension (PAH) and interstitial lung disease (ILD) are serious manifestations of systemic sclerosis (SSc). Previous reports suggest that PAH develops in approximately 7% of these patients and is a leading cause of death [1, 2]. Studies have examined exhaled nitric oxide (eNO) as a marker of pulmonary hypertension in SSc [3–9]. NO is derived from the amino acid arginine and synthesized by the enzyme NO synthase (NOS). NO is a potent vasodilator which stimulates the production of cyclic 3′5′-monophosphate (cGMP), resulting in smooth muscle relaxation [10]. The exact source of eNO is uncertain but likely represents a mixture of NO derived from the alveolar surface and from airway epithelial cells [11]. To date, studies examining the potential role of eNO as a marker in SSc-PAH have suggested decreased eNO or alveolar NO (*C*_*A*_NO), compared to individuals with SSc alone [3, 6, 8, 9]. In SSc-ILD, studies have suggested that eNO or *C*_*A*_NO is increased compared to control subjects, particularly if active alveolitis is present [3, 8, 12–15]. As a prognostic marker, *C*_*A*_NO levels may predict the occurrence of a 10% decrease in total lung capacity or forced vital capacity or death in patients with SSc [16].

This body of work suggests that exhaled nitric oxide may be a useful measure for use in SSc longitudinal observational studies or clinical trials. Indeed, there have been calls for novel methods to study SSc lung disease in clinical research [17]. However, important aspects of exhaled nitric oxide as a measure need to be ascertained prior to its implementation in the design of studies, including feasibility, the preferred method of measuring exhaled nitric oxide, and estimates of the magnitude of differences between cases and controls. The aim of this study was to evaluate exhaled nitric oxide as an outcome measure in SSc lung disease. The objective was to evaluate the discriminative validity of eNO in SSc lung disease. We also assessed the ability to recruit patients and conduct eNO measurements in the clinical setting. Given perioral skin tightening in SSc, we wanted to evaluate the feasibility of conducting these measurements in SSc subjects. We secondarily wanted to comparatively evaluate methods of measuring eNO to identify the preferred method. We evaluated the magnitude of differences across lung diseases (PAH, ILD, and both), and patient subsets (SSc, disease-control subjects (systemic lupus erythematosus (SLE), IPAH), and healthy-control subjects) to inform sample size and power estimates. The demonstration of feasibility, estimates of magnitude of differences between cases and controls, and demonstrable discriminative validity are all necessary prerequisites of a measure for its implementation as an outcome measure in clinical trials and observational studies.

## 2. Materials and Methods

### 2.1. Subjects

The University Health Network Pulmonary Hypertension Program (Toronto General Hospital, Toronto, ON, Canada) is the largest published longitudinal pulmonary hypertension cohort in Canada [18]. All patients undergo a standardized visit at least twice a year, including physical exam, laboratory testing, and investigations (CT thorax, echocardiogram, pulmonary function testing, serum BNP, cardiac catheterization, and six-minute walk test) as appropriate. The Toronto Scleroderma Program (Toronto Western Hospital, Mount Sinai Hospital, Toronto, ON, Canada) is the largest single-center SSc cohort in Canada [19]. All patients undergo a standardized visit every 6–12 months, including physical exam, laboratory testing, pulmonary function testing, and transthoracic echocardiography. Consecutive patients attending either program were screened by their physician or by the pulmonary function technician for study participation.

Subjects were included if they were >18 years, classified as SSc (American College of Rheumatology (ACR)-European League Against Rheumatism classification criteria for systemic sclerosis) [20] or SLE (ACR classification criteria for SLE) [21, 22], had mPAP > 25 mmHg on right-heart catheterization [23], and/or ILD based on a CT thorax ILD score >5% [24] and normal left ventricular function on echocardiogram. Subjects were excluded if they were pregnant, had HIV, congenital cardiac abnormalities, or had grade 2 left ventricular dysfunction or higher on echocardiogram.

### 2.2. Exhaled Nitric Oxide

Exhaled NO was measured using the Sievers GE 280i Nitric Oxide Analyser (Boulder, Colorado). Patients were instructed to inhale maximally (room air) and exhale against resistance to achieve continuous flow rates of 50, 100, 150, 200, and 250 mL/min. Patients had a visual marker indicating when they had achieved the desired flow rate. Exhaled NO was assessed for each flow rate on a separate exhalation. *F*_*E*_NO values at each flow rate are the mean of three plateau values on the *F*_*E*_NO time curves. The plateau values were determined by the Sievers' Analyser algorithm. Exhaled concentrations of NO at each flow rate were compared between each group and controls (Model 1). In addition, the two-compartment model of axial diffusion (CMAD) was used [7, 25–29]. The following calculation was used to partition alveolar (*C*_*A*_) versus conducting airway (*J*_awNO_′) components of the respiratory tract (Model 2):

(1) $$\begin{array}{c} V_{NO}=C_{A}\times V_{exh}+J_{awNO}^{\prime }=F_{E}NO\times V_{exh}, \end{array}$$

where *V*_NO_ is NO output (pL/s), *C*_*A*_ is steady state alveolar concentration of NO (ppb), *V*_exh_ is flow rate (mL/s), *J*_awNO_′ is total molar flux of NO in nanolitres/s (at an infinite *V*_exh_) in nL/s, *F*_*E*_NO is exhaled NO concentration (ppb), and Ppb is nL/L (1 × 10^−9^).

*C*_*A*_ was determined by calculating the slope of the line, and *J*_awNO_′ was determined by the *y*-intercept when multiple flow rates are assessed:

(2) CA=slope=VNO−JNOVexh=FENO−JNOVexhJawNO′=y−intercept=VNO−CA×Vexh.

A third approach (Model 3) using the trumpet model of axial diffusion (TMAD) corrects for the trumpet shape of the lungs (increasing surface area per unit volume) and the gas phase axial diffusion. In the initial derivation studies by Condorelli et al. [30], the alveolar concentration *C*_*A*_ was statistically lower, with *J*_awNO_′ being statistically higher compared to the CMAD. The correction factors in the trumpet model were

(3) CA=slope−y-intercept740 mL/sJawNO′=1.7×y-intercept.

### 2.3. BNP

Serum BNP measurements were obtained using the Bayer Centaur chemiluminescent assay (normal range ≤ 99.9 pg/mL).

### 2.4. CT Thorax

All participants underwent CT thorax. CT scans were reviewed by a blinded respirologist (JT). The extent of ILD (0%–100% using 5% intervals) was measured at 5 thoracic levels: the origin of the great vessels; the main carina; the pulmonary venous confluence; halfway between the third and fifth sections; and immediately above the right hemidiaphragm. The scores at each level were averaged to create a single score. This score was used to stage the severity of ILD using a validated SSc-ILD staging system [24]. A HRCT score ≤ 10% or HRCT of 11–30% (termed indeterminate) and FVC ≥ 70% was staged as limited disease. A HRCT score > 30% or HRCT of 11–30% and FVC < 70% was staged as extensive disease.

### 2.5. Data Administration

Connective tissue disease diagnosis, comorbidities, diagnostic tests, medications (prednisone, nonsteroidal anti-inflammatories), and smoking status were collected from the electronic health record or clinic chart(s) by a single abstractor. Data were double-entered into a computerized database.

### 2.6. Ethics, Consent, and Permissions

This study was approved by the Research Ethics Board of the University Health Network (reference number 12-5777 AE) [31]. Subjects provided written informed consent.

### 2.7. Statistical Analysis

Descriptive statistics were used to summarize the data. Exhaled NO was assessed using all three models. Pearson's product moment correlation coefficient and 95% confidence intervals (95% CI) were used to evaluate associations between eNO and serum BNP to hemodynamics, pulmonary function test parameters, and severity of ILD within and between groups. Subgroup analyses were conducted stratified by subset (SSc cases (SSc without lung disease, SSc-PAH, SSc-ILD, SSc-PAH, and ILD), disease-control subjects (SLE-PAH, IPAH), and healthy-control subjects). A *p* value of <0.05 was considered statistically significant. Analyses were conducted using RStudio (version 0.98.501).

## 3. Results

### 3.1. Study Subjects

Sixty subjects were recruited. There were 35 SSc cases and 25 control subjects. The case mix of SSc cases included SSc subjects without lung disease (*n* = 16), SSc-PAH (*n* = 7), SSc-ILD (limited) (*n* = 8), and SSc-ILD (extensive) (*n* = 4). The median CT score was 12% for subjects with limited involvement and 38% for subjects with extensive involvement. The median disease duration from diagnosis to study recruitment for SSc subjects was 9 (range 1–32) years. The disease-control subject case mix included SLE-PAH (*n* = 6) and IPAH (*n* = 9). There were 10 healthy controls. Subject characteristics are summarized in Table 1. Eighty-two percent of subjects were female. The SLE-PAH group was younger than the other groups (median age 35 years). Most subjects with SSc (89%) had limited disease. All SSc participants had a history of Raynaud's phenomenon and none had a history of renal crisis. Most had telangiectasia (78%), sclerodactyly (78%), and esophageal dysmotility (72%). In subjects with PAH, the median mPAP ranged from 38 to 44 mmHg and was similar between groups. The median LVEDP was also similar ranging from 6 to 9 mmHg. CT ILD scores for ILD subjects ranged between 8% and 50%.

### 3.2. Exhaled NO

Exhaled NO values measured at a single exhalation at flow rates ranging from 50 mL/s to 250 mL/s did not distinguish between groups. The CMAD model identified differences in alveolar NO (*C*_*A*_NO) and conducting airway (*J*_awNO_′) NO between groups. Healthy-control subjects had lower median (interquartile range (IQR)) alveolar NO (*C*_*A*_NO) than PAH patients (2.0 ppb (1.5, 2.5) versus 3.14 ppb (2.3, 4.0), *p* = 0.008) and specifically versus SSc-PAH patients 3.3 ppb (3.0, 5.7), *p* = 0.01) (Tables 2 and 3). SSc-ILD patients had significantly lower median *J*_awNO_′ values compared to controls (1009.5 versus 1342.1 ml*∗*ppb/s, *p* = 0.04). Compared to all subjects with PAH, subjects with extensive ILD appeared to have lower *C*_*A*_NO (3.14 versus 2.94, *p* = 0.34) and *J*_awNO_′ (1072.4 versus 921.6, *p* = 0.42). Compared to SLE-PAH subjects, SSc-PAH subjects appeared to have higher *C*_*A*_NO (4.29 versus 2.77, *p* = 0.13) and lower *J*_awNO_′ (950.3 versus 1177.7, *p* = 0.28). Exploratory analyses limited to female subjects found qualitatively similar findings but were not statistically significant (Table 3).

The TMAD model yielded multiple negative *C*_*A*_NO values, the interpretation of which is unclear (results not presented). This suggests that this modeling may not apply to patients with the diseases studied here.

**Figure 1:**
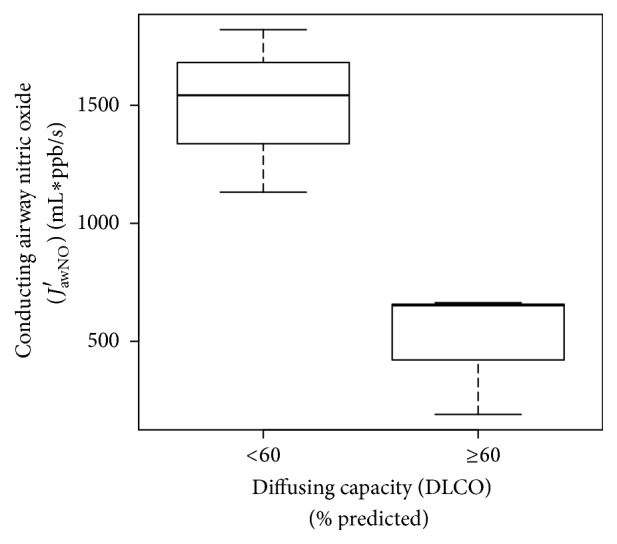
Relationship of conducting airway nitric oxide to diffusing capacity in SSc-PAH. Box plot illustrating increased conducting airway exhaled nitric oxide has discriminative validity in SSc-PAH subjects with reduced diffusing capacity.

Exploratory analyses evaluating the association of *C*_*A*_NO and *J*_awNO_′ values in relation to pulmonary function testing parameters, mean pulmonary artery pressure, and right ventricular systolic function were conducted. In SSc-PAH patients, increased *J*_awNO_′ correlated with a reduced DLCO (Pearson's *r*  −0.88 (95% CI −0.99, −0.26)). Patients with a DLCO less than 60% predicted had higher *J*_awNO_′ compared to those with a DLCO greater than or equal to 60% as illustrated in Figure 1.

There were no significant correlations between FVC, FEV1, and TLC with either alveolar NO (*C*_*A*_NO) or conducting airway (*J*_awNO_′) (Tables 4–7).

### 3.3. Age and Sex

We found no correlation between exhaled NO and age for all subjects and subgroups (Table 7).

### 3.4. Serum BNP

Median serum BNP values were higher in SSc-PAH patients than SSc patients without pulmonary involvement (358.8 versus 11.6 pg/ml, *p* = 0.01). No other significant differences were identified between groups.

## 4. Discussion

Exhaled NO is widely used as a noninvasive marker of airway inflammation in asthma; however research into its utility in scleroderma lung disease is less well developed and less well known [32]. In this study, eNO values were assessed using multiple models and demonstrated differences in exhaled alveolar NO (*C*_*A*_NO) and conducting airway NO (*J*_awNO_′) between groups, depending on the type of pulmonary pathology present. PAH subjects, particularly SSc-PAH subjects, appear to have higher exhaled alveolar NO than healthy subjects. In SSc-PAH subjects, increased conducting airway NO correlates with a reduced DLCO. However, SSc-ILD subjects had lower conducting airway NO than healthy subjects. BNP was higher in SSc-PAH subjects than SSc subjects without pulmonary involvement.

We found that median exhaled alveolar NO (*C*_*A*_NO) was highest in subjects with SSc. This aligns with the results of previous studies examining *C*_*A*_NO values in subjects with SSc [3, 7, 13–15, 33, 34]. The median *C*_*A*_NO value for the SSc-PAH group was significantly higher than that in control subjects. This demonstrates concurrent validity with the findings of two studies measuring *C*_*A*_NO in individuals with SSc-PAH [3, 14]. A possible mechanism for the observed increased *C*_*A*_NO may include decreased diffusion of NO into the pulmonary circulation due to reduced pulmonary capillary volume, destruction of the vascular bed, alveolar/capillary block in ILD, or ventilation-perfusion mismatch. This hypothesis is supported by negative correlations between *C*_*A*_NO and DLCO as reported by others [7, 35].

In SSc-PAH patients, increased conducting airway NO (*J*_awNO_′) correlated with a reduced DLCO. This difference in conducting airway NO (*J*_awNO_′) is illustrated in SSc-PAH patients with a DLCO greater than versus less than 60% predicted and supports the divergent validity of conducting airway NO testing [36]. The pathophysiologic relationship between conducting airway NO (*J*_awNO_′) and DLCO in SSc-PAH warrants further investigation, especially as DLCO is informative in the evaluation of PAH.

The exploratory analyses suggest that SLE-PAH subjects have lower alveolar NO and higher conducting airway NO than SSc-PAH subjects whereas alveolar NO is more comparable between SSc-PAH and IPAH subjects. This suggests that although SLE-PAH subjects have similar mean pulmonary artery pressure elevations on cardiac catheterization as SSc-PAH subjects, it may not be related to the same NO signaling abnormalities.

We explored the correlation between exhaled NO and age. We found no significant correlations with age across all the subgroups, suggesting a lack of age-dependent regulation of NO. We also explored differences in alveolar NO and conducting airway NO in solely female subjects, as there has been a suggestion that PAH pathology may be sex dependent. In our SSc-PAH cohort, we have previously demonstrated sex disparities in the frequency of PAH, time to PAH diagnosis, PAH disease duration, and SSc disease burden; however male sex did not independently impact SSc-PAH survival [37]. The results of the subgroup analysis in the current study remained qualitatively unchanged but were not statistically significant. This is likely related to a reduction in sample size and resultant power. The small sample size limits the precision around our estimates. However, the sample size was sufficient to obtain between group differences to inform sample size and power estimation for clinical trials. It is important to note that the study definition of ILD was based on the CT SSc-ILD system [24]. This may have resulted in the inclusion of subjects with mild ILD compared to studies based on pulmonary function tests alone.

The trumpet model of axial diffusion (TMAD) did not perform well in this study. It may be that this model is not applicable to connective tissue disease subjects, despite being used by others [9]. Models of NO excretion in the airway, including the TMAD model, are theoretical and may not apply to all disease states. The TMAD model corrects observed values for the flow-independent NO parameters based on the trumpet geometry of the airways and also on axial diffusion, which is diffusion from terminal airways to the alveolar region against the direction of exhalation flow. Application of the TMAD model resulted in a small proportion of negative values for CANO in our cohort, which is physiologically impossible. This suggested that this model is not applicable to SSc. The reasons for this are unknown, but, possibly, the distortion of lung architecture has changed airway geometry, while the alveolar capillary block due to fibrosis may have reduced the impact of axial diffusion.

Compared to *F*_*E*_NO, assessing conducting airway and alveolar NO may allow us to discriminate more effectively between symptomatic subjects with SSc and associated PAH and/or ILD. In the setting of asthma, eNO measurement is used to clarify the cause of symptoms where more than one factor may be contributing to symptoms (including anxiety, obesity). Where symptoms and inflammation are discordant, eNO measurement provides useful information [38]. The CMAD models have provided evidence to support the presence of different microenvironments for NO production and metabolism in the conducting airways compared to the alveoli. This may ultimately be helpful in furthering our knowledge of the pathophysiology of PAH and ILD in SSc and also help to build on the hypothesis that PAH generally reflects a state of “NO deficiency.” NO is currently targeted with phosphodiesterase type 5 inhibitors and stimulators of guanylate cyclase, yielding hemodynamic and symptomatic improvement in individuals with PAH [39, 40]. Improving our understanding of the relationship between eNO and PAH in an etiology-specific manner is important, as the underlying balance between alveolar, vascular, and conducting airway eNO appears to vary with specific causes of PAH which may have implications for clinical management. However, currently, the performance of eNO determination at multiple flow rates is experimental and only suitable for academic centers with the equipment and knowledge required to perform these measures. The integration of eNO as a valuable marker in scleroderma pulmonary disease will require further validation and dissemination of knowledge.

## 5. Conclusion

We have demonstrated feasibility (ability to recruit and conduct these measurements in SSc subjects); identified that CMAD modeling is preferred in SSc subjects; and generated pilot data for the magnitude of differences across lung diseases, patient subsets, and healthy controls, to base future sample size and power estimates. Our data supports discriminative validity of eNO in SSc lung disease. Our demonstration of feasibility, estimates of magnitude of differences between cases and controls, and demonstrable discriminative validity provide necessary prerequisite evaluation of a novel measure prior to its implementation as an outcome measure in clinical trials and observational studies of SSc lung disease.

## Figures and Tables

**Table 1:** Summary of subject characteristics.

	SSc *n* = 16	SSc-PAH *n* = 7	SLE-PAH *n* = 6	SSc-ILD limited *n* = 8	SSc-ILD extensive *n* = 4	IPAH *n* = 9	Control *n* = 10
Female sex (%)	16 (100%)	5 (71%)	7 (100%)	7 (88%)	3 (75%)	7 (78%)	5 (50%)
Age in years (median)	51	51	37	54.5	56.5	41.0	33.5
Limited cutaneous subtype (%)	15 (94%)	5 (71%)	NA	7 (88%)	2 (50%)	NA	NA
*Manifestations (%)*							
Calcinosis	6 (38%)	2 (29%)	NA	1 (13%)	2 (50%)	NA	NA
Raynaud's phenomenon	16 (100%)	7 (100%)	NA	8 (100%)	4 (100%)	NA	NA
Esophageal dysmotility	13 (81%)	6 (86%)	NA	5 (63%)	4 (100%)	NA	NA
Sclerodactyly	12 (75%)	6 (86%)	NA	5 (63%)	4 (100%)	NA	NA
Telangiectasia	13 (81%)	7 (100%)	NA	5 (63%)	3 (75%)	NA	NA
Renal crisis	0	0	NA	0	0	NA	NA
Abnormal nailfold capillaries	5 (31%)	5 (71%)	NA	3 (38%)	2 (50%)	NA	NA
Digital ulcers	3 (19%)	5 (71%)	NA	2 (25%)	2 (50%)	NA	NA
ScL-70 antibody	1 (6%)	0	NA	2 (25%)	2 (50%)	NA	NA
Anti-centromere antibody	6 (38%)	1 (14%)	NA	2 (25%)	1 (25%)	NA	NA
*Hemodynamics*							
mPAP mmHg (median, IQR)	NA	40 (37–48)	42 (39–48)	NA	38 (34–40)	44 (42–53)	NA
LVEDP mmHg (median, IQR)	NA	8 (4–10)	6 (6–11)	NA	6 (4–8)	9 (7–13)	NA
*Comorbidities (%)*							
Asthma	2 (13%)	0	2 (29%)	1 (13%)	0	2 (22%)	0
COPD	0	0	0	0	0	0	0
OSA	2 (13%)	0	1 (14%)	1 (13%)	0	1 (11%)	0
Systemic hypertension	4 (25%)	1 (14%)	1 (14%)	2 (25%)	2 (50%)	3 (33%)	0
Atrial fibrillation	0	0	0	0	1 (25%)	0	0
CAD	1 (6%)	1 (14%)	0	0	0	1 (11%)	0
*Smoking history (%)*							
Current	2 (13%)	1 (14%)	0	0	0	0	NA
Former	6 (38%)	3 (43%)	2 (29%)	2 (25%)	2 (50%)	3 (33%)	NA
*Medication (%)*							
NSAID	2 (13%)	2 (29%)	2 (29%)	5 (63%)	0	2 (22%)	0
Prednisone	0	2 (29%)	5 (71%)	3 (38%)	2 (50%)	0	0
Inhaled corticosteroids	1 (6%)	0	0	1 (13%)	0	1 (11%)	0
*Pulmonary function tests*							
FEV1% predicted (median)	92.0	95.0	78.0	85.0	71.0	87.5	NA
FVC% predicted (median)	97.0	95.0	81.0	89.0	65.0	90.5	NA
TLC% predicted (median)	96	104	83	95	74	90	NA
DLCO% predicted (median)	72.0	48.5	73.5	58.0	65.5	70.5	NA

mPAP: mean pulmonary artery pressure, LVEDP: left ventricular end-diastolic pressure, COPD: chronic obstructive pulmonary disease, OSA: obstructive sleep apnea, CAD: coronary artery disease, NSAID: nonsteroidal anti-inflammatories, FEV1: forced expiratory volume in one second, FVC: forced vital capacity, TLC: total lung capacity, DLCO: diffusing capacity.

**Table 2:** Comparison of exhaled nitric oxide values between groups.

Group	*C*_*A*_NO Ppb (median)	*J*_awNO_′ nL/s (median)
*All subjects*		
All ILD versus controls	2.34 versus 2.03	**1009.5 **versus** 1342.1**
All PH versus controls	**3.14 **versus** 2.03**	1066.8 versus 1342.1
*Female subjects*		
All ILD versus controls	2.30 versus 2.47	1065 versus 1196
All PH versus controls	3.02 versus 2.47	938 versus 1196

*Note*. Bold denotes significant finding, Ppb = parts per billion.

**Table 3:** Median *C*_*A*_NO and *J*_awNO_′ by subgroup.

	SSc	SSc-PAH	SSc-ILD	SSc-PAH + ILD	SLE-PAH	IPAH	Control
*All subjects*							
*C*_*A*_NO Ppb median	4.00	**3.30**	2.34	2.84	2.80	3.32	**2.03**
*J*_awNO_′ nL/s median	988.9	721.0	**1009.5**	1032.0	1138.3	952.0	**1342.1**
*Female subjects*							
*C*_*A*_NO Ppb median	4.00	3.30	2.34	2.09	2.80	3.32	2.47
*J*_awNO_′ nL/s median	988.9	662	1009.5	1112.0	1138.3	924	1196

Bold denotes significant finding, Ppb = parts per billion.

**Table 4:** Correlation between alveolar NO (*C*_*A*_NO) and pulmonary function testing.

Group	TLC	FEV1	FVC	DLCO
SSc	−0.32 (−0.69, 0.16)	−0.40 (−0.73, 0.08)	−0.39 (−0.72, 0.09)	−0.44 (−0.76, 0.03)
SSc-PAH	0.39 (−0.62, 0.91)	0.06 (−0.73, 0.78)	−0.26 (−0.85, 0.62)	0.35 (−0.65, 0.90)
SLE-PAH	−0.13 (−0.91, 0.84)	0.75 (−0.39, 0.98)	0.83 (−0.19, 0.99)	−0.14 (−0.91, 0.85)
SSc-ILD	−0.32 (−0.98, 0.93)	−0.25 (−0.97, 0.94)	0.25 (−0.94, 0.98)	−0.95 (−1.0, 0.13)
IPAH	−0.25 (−0.88, 0.70)	−0.49 (−0.89, 0.32)	−0.40 (−0.86, 0.42)	0.30 (−0.67, 0.89)

None of the correlations were significant.

**Table 5:** Correlation between conducting airway NO (*J*_awNO_′) and pulmonary function testing.

Group	TLC	FEV1	FVC	DLCO
SSc	0.46 (−0.01, 0.76)	0.32 (−0.17, 0.68)	0.05 (−0.43, 0.50)	0.05 (−0.42, 0.51)
SSc-PAH	−0.79 (−0.98, 0.04)	−0.41 (−0.89, 0.50)	−0.33 (−0.87, 0.56)	**−0.88 (−0.99, −0.26)**
SLE-PAH	0.42 (−0.73, 0.95)	−0.44 (−0.95, 0.72)	−0.10 (−0.90, 0.85)	0.58 (−0.62, 0.97)
SSc-ILD	0.23 (−0.94, 0.98)	0.08 (−0.95, 0.97)	−0.47 (−0.99, 0.90)	0.59 (−0.86, 0.99)
IPAH	−0.25 (−0.88, 0.70)	−0.49 (−0.89, 0.33)	−0.40 (−0.86, 0.42)	0.30 (−0.68, 0.89)

Bold denotes significant correlations.

**Table 6:** Correlation between alveolar NO, conducting airway NO, and mean pulmonary artery pressure.

Group	mPAP to *C*_*A*_NO	mPAP to *J*_awNO_′
SSc-PAH	0.42 (−0.48, 0.89)	0.34 (−0.56, 0.87)
SLE-PAH	0.47 (−0.55, 0.93)	0.36 (−0.64, 0.91)
IPAH	−0.06 (−0.70, 0.63)	0.38 (−0.37, 0.83)

*Note*. Only subjects with PAH underwent right heart catheterization.

**Table 7:** Correlation between exhaled NO and age.

Group	Alveolar NO (*C*_*A*_NO) *r* (95% CI)	Conducting airway NO (*J*_awNO_′) *r* (95% CI)
All subjects	0.16 (−0.10, 0.39)	0.29 (−0.08, 0.59)
SSc	0.25 (−0.24, 0.64)	0.12 (−0.36, 0.56)
SSc-PAH	−0.58 (−0.93, 0.30)	0.48 (−0.43, 0.91)
SSc-ILD	0.78 (−0.72, 1.00)	−0.92 (−1.0, 0.33)
SLE-PAH	−0.56 (−0.94, 0.46)	−0.21 (−0.87, 0.73)
IPAH	−0.60 (−0.90, 0.11)	0.13 (−0.58, 0.73)
Healthy controls	−0.07 (−0.67, 0.59)	0.41 (−0.29, 0.83)

## Abbreviations

*C*_*A*_: Steady state alveolar concentration of NO (nL/L)
*C*_*A*_NO: Exhaled alveolar nitric oxide
cGMP: Cyclic 3′5′-monophosphate
CMAD: Compartment model of axial diffusion
eNO: Exhaled nitric oxide
*F*_*E*_NO: Exhaled NO concentration (ppb)
ILD: Interstitial lung disease
IPAH: Idiopathic pulmonary arterial hypertension
*J*_awNO_′: Total molar flux of NO in nanolitres/s (at an infinite Vexh) in nL/s
NOS: Nitric oxide synthase
PAH: Pulmonary arterial hypertension
Ppb: nL/L (1 × 10^−9^)
SLE: Systemic lupus erythematosus
SSc: Systemic sclerosis
TMAD: Trumpet model of axial diffusion
*V*_exh_: Flow rate (mL/s)
*V*_NO_: NO output (pL/s).
